# Time related variations in stem cell harvesting of umbilical cord blood

**DOI:** 10.1038/srep21404

**Published:** 2016-02-24

**Authors:** Gianluigi Mazzoccoli, Giuseppe Miscio, Andrea Fontana, Massimiliano Copetti, Massimo Francavilla, Alberto Bosi, Federico Perfetto, Alice Valoriani, Angelo De Cata, Michele Santodirocco, Angela Totaro, Rosa Rubino, Lazzaro di Mauro, Roberto Tarquini

**Affiliations:** 1 Department of Medical Sciences, Division of Internal Medicine and Chronobiology Unit, IRCCS “Casa Sollievo della Sofferenza”, S.Giovanni Rotondo (FG), Italy; 2 Apulia Cord Blood Bank, IRCCS “Casa Sollievo della Sofferenza”, S.Giovanni Rotondo (FG), Italy; 3 Unit of Biostatistics, IRCCS “Casa Sollievo della Sofferenza”, S.Giovanni Rotondo (FG), Italy; 4 Computing Unit, IRCCS Scientific Institute and Regional General Hospital “Casa Sollievo della Sofferenza”, S.Giovanni Rotondo (FG), Italy; 5 Department of Clinical and Experimental Medicine, Unit of Haematology, School of Medicine, University of Florence, Florence, Italy; 6 Department of Clinical and Experimental Medicine, School of Medicine, University of Florence, Florence, Italy; 7 Interinstitutional Department for Continuity of Care of Empoli, School of Medicine, University of Florence, Florence, Italy

## Abstract

Umbilical cord blood (UCB) contains hematopoietic stem cells and multipotent mesenchymal cells useful for treatment in malignant/nonmalignant hematologic-immunologic diseases and regenerative medicine. Transplantation outcome is correlated with cord blood volume (CBV), number of total nucleated cells (TNC), CD34+ progenitor cells and colony forming units in UCB donations. Several studies have addressed the role of maternal/neonatal factors associated with the hematopoietic reconstruction potential of UCB, including: gestational age, maternal parity, newborn sex and birth weight, placental weight, labor duration and mode of delivery. Few data exist regarding as to how time influences UCB collection and banking patterns. We retrospectively analyzed 17.936 cord blood donations collected from 1999 to 2011 from Tuscany and Apulia Cord Blood Banks. Results from generalized multivariable linear mixed models showed that CBV, TNC and CD34+ cell were associated with known obstetric and neonatal parameters and showed rhythmic patterns in different time domains and frequency ranges. The present findings confirm that volume, total nucleated cells and stem cells of the UCB donations are hallmarked by rhythmic patterns in different time domains and frequency ranges and suggest that temporal rhythms in addition to known obstetric and neonatal parameters influence CBV, TNC and CD34+ cell content in UBC units.

Umbilical cord blood (UCB) is blood that is enclosed in the placenta and the adjoined umbilical cord following delivery. UCB contains hematopoietic stem cells and to a lesser extent multipotent mesenchymal cells^1^,^2^. UCB can be used as hematopoietic stem cell source for transplantation and is increasingly utilized in the treatment of malignant and nonmalignant hematologic and immunologic diseases, being, in some cases, an alternative to bone marrow transplantation^3^. Recent work has highlighted the potential role of UCB in regenerative medicine^4^,^5^. When compared to hematopoietic stem cells deriving from bone marrow, UCB provides rapid availability of HLA-typed transplants that can be stored frozen until use, lower risk of graft versus-host disease regardless of HLA mismatch and no risk or pain for the donor during sample collection^6^. Shortcomings include the restricted number of stem cells and nucleated cells in UCB compared to marrow transplants, and the unfeasibility of using the donor for a second transplantation or donor lymphocyte infusion^7^. The outcome of transplantation is highly correlated with the number of total nucleated cells (TNCs), CD34+ cells and colony forming units (CFUs) in UCB donations. Numbers of TNCs in the count range of 2 × 10^8^ to 4 × 10^8^ per kilogram of body weight are considered adequate for a realistic engraftment, whereas lower numbers are associated with more protracted aplasia and poorer survival, primarily driven by an increased risk of infectious complications^7^,^8^. A number of studies have analyzed maternal and neonatal factors associated with hematopoietic reconstruction potential in UCB units, providing evidence of a role for gestational age, maternal parity, sex and birth weight of the newborn, placental weight, labor duration and mode of delivery^9^,^10^,^11^,^12^,^13^,^14^.

Currently, there is a paucity of data regarding the temporal dimension of UCB collection and banking patterns as to their impact on the efficacy of stem cell harvesting^15^,^16^. Many biological phenomena show temporal variations that may occur randomly or rhythmically. The rhythmicity of biological phenomena may range from seconds to years, and natural rhythms may be characterized by a great variety of frequency ranges and time domains. The most frequent biological rhythms are temporally determined by the geophysical light-darkness alternation due to the Earth’s rotation around its axis, having a period of approximately 24 hours. These are defined as circadian from the latin *circa*, about, and *dies*, a day. Interestingly, circadian haematopoietic stem cell release in the bloodstream under steady-state conditions is modulated by photic cues derived from the geophysical light/dark cycle^17^. Another temporal dimension of biological rhythmicity is determined by astronomical cues, such as: the Earth’s yearly revolution around the Sun (circannual), with the inclination of the terrestrial axis that leads to seasonality influencing many biological processes, including hormone secretion^18^; the lunar phases related to the Moon’s orbit around the Earth (circatrigintan), which influence mammals^19^,^20^ and environmental niches of tidal species^21^; and geomagnetic activity (circannual) or solar activity (circadecennial), which seem to influence human physiological processes and pathophysiological phenomena^22^,^23^.

In this study, we evaluated the time related variations of cord blood volume (CBV), TNC and CD34+ cell content of UCB units in relation to the obstetric, neonatal and collection factors that influence the volume and haematopoietic cell content of UCB donations. These factors were then related to time scales using retrospectively data derived from cord blood donations in the Tuscany and Apulia Cord Blood Banks.

## Matherials and Methods

### Ethics Statement

The study was approved by the ethical board of IRCCS Scientific Institute and Regional General Hospital “Casa Sollievo della Sofferenza”, S. Giovanni Rotondo (FG), Italy. The data were analyzed anonymously and all investigations were conducted according to the principles expressed in the Declaration of Helsinki. Maternal written informed consent to proceed with the donation was obtained by all subjects.

### Cord blood collection and testing

The obstetric services of two Italian regions, Tuscany (43°25′N, 11°01′E) and Apulia (41°27′N, 15°34′E), participated in the program from the beginning of 1999 to the end of 2011. The only a priori exclusion criteria were maternal human immunodeficiency virus or hepatitis B virus infections, known to affect the infant’s hematopoietic system, as well as increase the risk of stillbirth or neonatal death. Maternal history was reviewed for complications of pregnancy, labor and delivery, as determined by the mother’s obstetrician, as well as the estimated date of confinement. Maternal medical history and family history of genetic diseases were determined by interview, with an emphasis on the presence of risk factors for exposure to blood transmissible infectious agents and risks of genetic diseases that affect the hematopoietic system. All mothers who participated in the program were Caucasian. The infant’s medical chart was examined for sex, birth weight, 5-minute Apgar score, and overall health prior to hospital discharge. For this study we evaluated the following variables: demographic characteristics, route of delivery, infant gestational age, length of labor, duration of ruptured membranes, newborn weight and placental weight. Following maternal consent and the approval of the Ethical Committees and the hospital’s institutional review boards, eligible donated UCB units were retained in an inventory for unrelated patients whose diseases would require a bone marrow replacement. UCB units were collected in utero and processed as described elsewhere^24^. Briefly, UCB units were collected by dedicated staff at any time of day, seven days per week, from pregnant women who reached at least gestation week 37. Appropriate counseling was provided, and informed consent obtained, for UCB donation from pregnant women and their partners at gestation week 35. We also carefully collected the couple’s history, focusing on genetic, immunological and infectious diseases on both the maternal and paternal sides (anamnestic criteria). UCB units were not collected or were discarded on the basis of the following maternal and foetal obstetric criteria: *(i)* ruptured membrane more than 12 hours before the delivery, *(ii)* maternal fever >38 °C, *(iii)* meconium stained amniotic fluid, *(iv)* too quick expulsion of the placenta, *(v)* dystocia, preeclampsia, placental abruption, *(vi)* low Apgar score at birth (<7 at 1 minute and <5 at 5 minutes), *(vii)* low birth weight (<2.600 g), and *(viii)* congenital malformations. Sixty seconds after spontaneous delivery or elective cesarean section, the umbilical cord was clamped and cleaned with povidone-iodine solution, and the most distant possible venipuncture site (closest to the clamp) was cleaned for 15 seconds with povidone-iodine solution and then with alcohol. The placenta and its attached umbilical cord were weighed after expulsion. The UCB was collected from the umbilical vein with a 12.5-gauge needle connected to a sterile 150 mL bag (Maco Pharma S.A. Laboratoires Pharmaceutiques, France), containing 21 mL of phosphate-citrate dextrose anticoagulant. Blood was drained into the collection bag by gravity until blood flow stopped, which was monitored by periodically weighing the collection bag. Milking of the umbilical cord was performed and the uterus was gently massaged during the last period of the collection in order to increase the blood flow. After collection the UCB was sent to the Cord Blood Bank of each collection center along with the anamnestic form and six samples of maternal blood. Following an initial laboratory check, all material was sent within 24–48 hours to the UCB bank, where the units were processed for cryopreservation. Cord blood units were characterized, processed and cryopreserved within 48 hours of collection. Our threshold for accepting cord blood for banking was a TNC count of 8 × 10^8^ (this value was elevated to 1.2 × 10^9^ from July 1, 2011). Before cryopreservation, volume reduction, blood cultures (to verify sterility), CD34+ cell counts, and plating for CFU assays were performed. After a few weeks the UCB bank reported the laboratory results. The laboratory criteria for excluding UCB from banking (biological criteria) were: *(i)* low cellularity (<8 × 10^8^), *(ii)* weight of the UCB unit bag <80 g, *(iii)* presence of blood clots, *(iv)* damage to the UCB bag, and *(v)* infections of the blood collected during the delivery. The last check of the units before definitive banking was performed 6 months after delivery, when it was determined whether the mother or infant has any clinical problems.

### Statistical Methods

Baseline maternal, newborn and cord blood characteristics were reported as means ± standard deviation (and medians along with minimum-maximum range), or frequencies and percentages for continuous and categorical variables, respectively. Data analysis was performed without pre-set thresholds. Normal distribution assumption was checked by means of Q-Q plot, Shapiro-Wilks and Kolmogorov-Smirnov tests. The Spearman Rank-Order Correlation Coefficient was calculated over the whole sample between the values of Apgar score at 1 and 5 minutes and CBV, TNC content, and CD34 + cell content. Generalized linear mixed models, accounting for clustering due to collector effect, were estimated to assess the influence of the time of birth and baseline clinical variables on CBV, TNC and CD34+ cell outcomes, separately. Specifically, CBV (log-transformed values) was modeled using a linear mixed model, whereas TNC and CD34+ cell outcomes were modeled using negative binomial models, with random intercepts. All the models included the following covariates: the categorical time variables, namely year, month, day and hour of newborn’s birth, as well as maternal age, gestational age, sex and birth weight of the newborn and route of delivery.

Adjusted means of CBV, TNC and CD34+ cell outcomes were further estimated for each level of categorical time variables. Comparisons of adjusted means between Tuscany and Apulia Cord Blood Banks were performed, estimating specific statistical contrasts from models.

The overall contribution provided by all covariates included in the multivariable model was estimated by the total R^2^ statistic, whereas the marginal contribution provided by each covariate (when all others are already included into the model) was estimated by the partial R^2^ coefficient. Mean daily deliveries were estimated and compared using the Poisson model, thereby accounting for overdispersion. The results were adjusted for multiple hypothesis testing.

Moreover, to assess the time related variations of CBV, TNC and CD34+ cell levels over years, months, days and hours, cosinor models with linearized coefficients, which best fit the previously estimated adjusted means, were estimated for each outcome within each time domain^25^. For each cosinor model, all possible ranges of fundamental time period length was evaluated and the one that achieved the minimum Akaike’s Information Criterion was chosen. Estimates of MESOR and harmonic-specific semi-amplitude and acrophase were derived from inverse transformations of the corresponding linear parameters estimates, applying the Delta method to derive their approximate standard errors.

A *p* value < 0.05 was considered statistically significant. All statistical analyses were performed using SAS Software Release 9.3 (SAS Institute Inc., Cary, NC, USA).

## Results

Maternal, newborn, placental, and obstetric characteristics for the 17.936 deliveries and cord blood donations are summarized in Table 1 (mean ± SD, median, range). Raw means in different time domains are rendered in supplemental Figs 1–3. Considering the whole sample, the collected CBV mean was 71.12 ± 28.40 ml (median: 68.57 ml, range: 10–231.43 ml), the mean content of TNCs was 983.57 ± 465.89 (median: 906.67, range: 10.30–4914.67), the mean content of CD34+ × 10^6^ was 3.51 ± 3.08 (median: 2.90, range: 0.01–33.27), and the mean CFU was 88.15 ± 63.96 (median: 71.25, range: 1.00–550.00). The Tuscany CBV mean was 62.00 ± 26.24 (median: 59.00, range: 10.00–214.47), and 84.25 ± 26.18 (median: 81.00, range: 19.05–231.43) in Apulia. The Tuscany mean TNC content was 1128.44 ± 465.63 (median: 1052.00, range: 10.30–4359.60), and 875.96 ± 436.00 (median: 797.95, range: 36.00–4914.67) in Apulia. The mean CD34+ × 10^6^ content was 2.49 ± 3.64 (median: 0.92, range: 0.01–33.27) in Tuscany, and 3.78 ± 2.85 (median: 3.13, range: 0.07–30.83) in Apulia. The mean CFU in Tuscany was 52.40 ± 36.50 (median: 46.50, range 1.00–538.17) and 125.05 ± 65.31 (median: 110.50, range 6.05–550.00) in Apulia. Over the whole sample, the mean Apgar score at 1 minute was 8.80 ± 1.24 (median 9, range: 1–9), being 9.01 ± 1.46 (median 9, range: 1–10) in Tuscany and 8.51 ± 0.76 (median: 9, range: 1–10) in Apulia. Over the whole sample, the mean Apgar score at 5 minutes was 9.56 ± 0.64 (median 9, range:1–10), being 9.67 ± 0.64 (median 9, range:1–10) in Tuscany and 9.39 ± 0.60 (median:9, range:1–10) in Apulia. Estimated adjusted means from multivariable generalized linear mixed models were statistically different between Apulia and Tuscany (all *p* < 0.001). When the Spearman Rank-Order Correlation Coefficient was calculated over the whole sample, there was a positive correlation between the values of Apgar score at 1 and 5 minutes (Rs = 0.531, *p* < 0.0001), and a negative correlation between the Apgar score values at 1 minute and CBV (Rs = −0.114, *p* < 0.0001), TNC content (Rs = −0.0526, *p* < 0.0001), and CD34+ cell content (Rs = −0.343, *p* < 0.0001). Likewise the Apgar score value at 5 minute was correlated negatively with CBV (Rs = −0.134, *p* < 0.0001), TNC content (Rs = −0.0677, *p* < 0.0001) and CD34+ cell content (Rs = −0.373, *p* < 0.0001).

In Table 2 are reported *p*-values for associations (as well as the total and partial R^2^) between CBV, TNC, CD34+ cells and time of birth along with baseline clinical variables from generalized linear mixed models. The results pinpointed the relevance of a covariate for the generalized linear model and clearly showed that year of birth, weight, sex, gestational age and mode of delivery were the only statistically significant covariates associated both to CBV and TNC outcomes (all *p* < 0.001). As for CD34+ cells, significant covariates were: year (*p* < 0.001), month (*p* = 0.021), hour of birth (*p* < 0.001), gestational age (*p* < 0.001), and infant’s birth weight (*p* < 0.001). Interestingly, the newborn’s weight was the covariate accounting for the greatest contribution to total variance, especially for TNC counts (partial R^2^ = 0.557).

In addition, the mean number of daily deliveries was significantly greater in Apulia than in Tuscany (*p* < 0.001). Indeed, the mean number of deliveries/day in Apulia was 5.13 (median: 5, interquartile range: 2–8, minimum-maximum range: 1–17), whereas in Tuscany it was 3.64 (median: 3 deliveries/day, interquartile range: 2–5, minimum-maximum range: 1–15).

**Figure 1:**
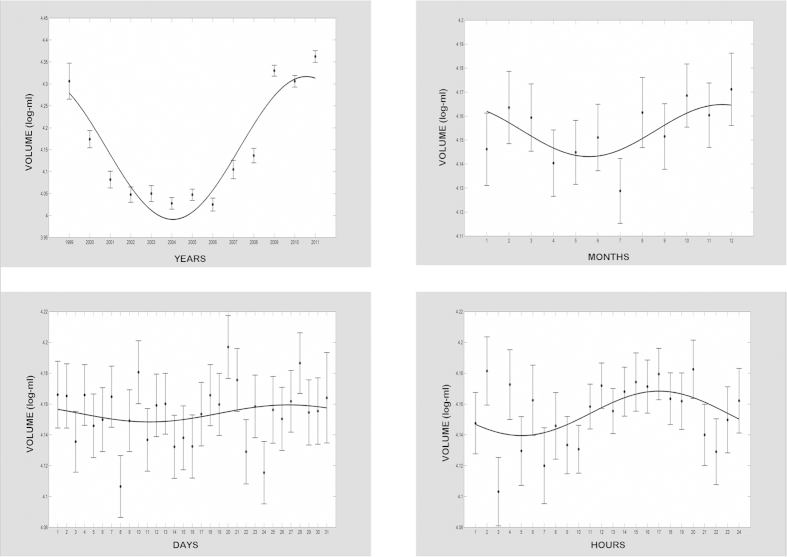
*x–y* plots showing from top to bottom the fitted cosine curves (continuous lines) superimposed on adjusted means and standard error of log-transformed values (ovals) and variation patterns in different time domains of volume in the cord blood donations in Tuscany and Apulia Cord Blood Banks from 1999 to 2011.

**Figure 2:**
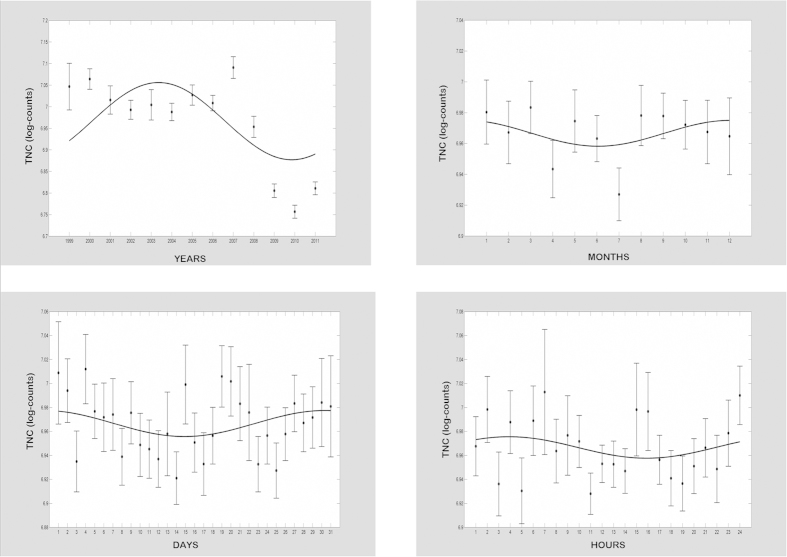
*x–y* plots showing from top to bottom the fitted cosine curves (continuous lines) superimposed on adjusted means and standard error of log-transformed values (ovals) and variation patterns in different time domains of total nucleated cells (TNCs) in the cord blood donations in Tuscany and Apulia Cord Blood Banks from 1999 to 2011.

**Figure 3:**
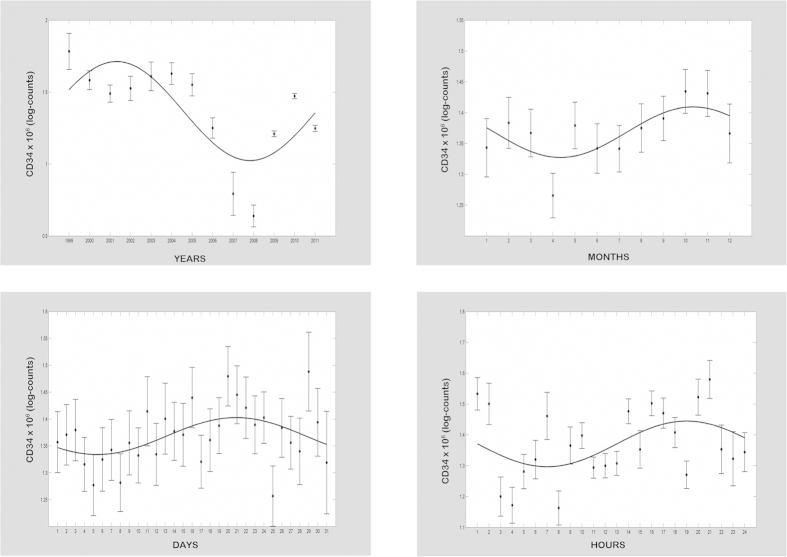
*x–y* plots showing from top to bottom the fitted cosine curves (continuous lines) superimposed on adjusted means and standard error of log-transformed values (ovals) and variation patterns in different time domains of CD34+ progenitor cells in the cord blood donations in Tuscany and Apulia Cord Blood Banks from 1999 to 2011.

CBV, TNC and CD34+ cell content of UCB units showed time related variations (Table 3 and Figs 1, 2, 3). The mean CBV differed over the 12 years of the study, showing a rhythmic pattern and a periodicity of approximately 10 years (*p* < 0.001) with the acrophase in the year 2011, superimposed on a circasemiannual rhythmicity (*p* < 0.001), a circasemitrigintan rhythm (*p* < 0.001), and a 24-h periodicity (*p* < 0.001), with the acrophase at 17:00 h. The content of TNCs showed circadecennial periodicity (*p* < 0.001) with the acrophase in the year 2003, superimposed on a circasemiannual rhythmicity (*p* < 0.001), circasemitrigintan periodicity (*p* < 0.001) and infradian pattern with a period of 8 hours (*p* < 0.001). The content of CD34+ progenitor cells showed circadecennial periodicity (*p* < 0.001) with the acrophase in the year 2001, superimposed on a circannual rhythm with the acrophase in October (*p* < 0.001), a rhythmic circatrigintan pattern with the acrophase in the fourth week (*p* < 0.001), and a circadian rhythm with the acrophase at 20:00 h (*p* < 0.001).

## Discussion

The physiology of living beings is characterized by periodic oscillations of processes and functions, in turn creating an array of biological rhythms that bring about the interaction of multicomponent systems^26^,^27^,^28^,^29^,^30^,^31^,^32^. The 24-h periodicity overlaps with rhythmic events that may occur at shorter intervals, such as pulsatile secretion of hormones and cyto/chemokines. This intersects with oscillations characterized by more extended periods, such as menstrual changes in females or the oscillation of significant biological regulators in the blood stream^33^.

The results of our retrospective analysis show that the temporal dimension influences the volume of cord blood collected, as well as the content of TNC and CD34+ cells in UBC units. Our results also confirm the importance of several obstetric and neonatal parameters, previously noted as crucial determinants of hematopoietic regenerative capacity in UCB donations^2^,^12^,^34^,^35^,^36^,^37^. Accordingly, the volume of cord blood collected and TNC content were significantly associated with the year of birth, gestational age and route of delivery, as well as newborn’s gender and weight. CD34+ cell content was associated with year, month and hour of birth, as well as gestational age and newborn’s birth weight. Furthermore, CBV as well as TNC and CD34+ cell contents, were negatively correlated with Apgar score at 1 and 5 minutes. This result corroborates the findings reported in previous articles showing that the volume of cord blood, the count of TNCs and the stem cell harvesting in UCB are significantly affected by perinatal factors related to stress and hypoxia, such as mode of delivery, gestational age, birth weight, gender, UCB pH, maternal fever and notably Apgar score values^1^,^2^,^3^,^4^,^5^,^6^,^7^,^8^,^9^,^10^,^11^,^12^,^13^,^14^.

In regard to the interactions of time related variations of CBV, TNC and CD34+ cell content of UCB units with maternal and neonatal predictors of stem cell content, our analysis evidenced rhythmic patterns with different frequency ranges. CBV changed over the 13 years of the study, showing a rhythm characterized by a time interval of approximately 10 years, suggesting a circadecennial periodicity with the acrophase in the year 2011. CBV showed variations in other time domains, in particular: rhythmic changes with lower values from May to August, suggesting a circasemiannual periodicity; a circasemitrigintan periodicity; and a clear 24-h periodicity, with the zenith in the afternoon and the nadir in the early morning. On the other hand, TNCs showed circadecennial periodicity with the acrophase in the year 2003 and with rhythmic patterns in half-yearly, half-monthly and infradian temporal domains. The content of CD34+ cells showed: circadecennial periodicity, with the acrophase in the year 2001; a circannual rhythm, with the lowest values between April and August and the acrophase at the end of October; a circatrigintan pattern with the acrophase at the end of the month; and a circadian rhythm with the acrophase in the evening.

Considering that the methods did not differ between the two centers, did not change during the study period and that UCB was collected 24 hours a day and 7 days a week, our results convincingly show that the temporal dimension influences the content of cord blood hematopoietic progenitors. Much larger year-to-year changes characterized the fluctuations of CBV, TNC, and CD34+ cell content when compared to the other time-scales, with an anti-phasic pattern of circadecennial periodicity for CBV compared to TNC and CD34+ cell content. Our data are in agreement with previous reports describing time related changes of stem cell content in cord blood donations, showing seasonal variations with lower values between May and August as well as circadian oscillation with the lowest values reported during the day and the highest values during the night^15^,^16^. The exploitation of estimated adjusted means from a generalized linear model which used log-transformed data distinctively avoided the impact of confounders such as collector experience and laboratory technical expertise and effectively rendered the actual biological phenomena, without the nosiness of confounding factors.

Previous work in humans and animals showed that the fluctuations of haematopoietic stem cells and their progenitors in the bloodstream are characterized by a roughly 24-hour cycle driven by circadian genes of mouse and human bone marrow CD34+ cells^38^,^39^,^40^, with the zenith of haematopoietic stem cells release occurring during the resting period in the mouse and in the evening in humans^17^,^41^,^42^,^43^,^44^. Such experimental work indicates that the trafficking of haematopoietic stem cells is tightly controlled by the circadian clock circuitry, implying a crucial role for genetic, hormonal (glucocorticoids and melatonin) and neural factors (sympathetic nervous system signalling), and suggesting a role for maternal factors passing over the placenta^45^,^46^,^47^,^48^,^49^.

**Figure 4:**
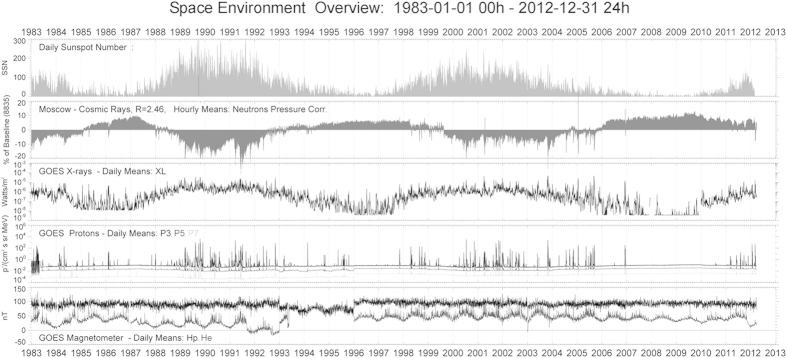
Data of solar flares, solar proton events (SPE), and geomagnetic activity measured with the space environment monitor system onboard the Geostationary Operational Environmental Satellites (GOES). Data of daily proton fluencies and SPE from the National Geophysical Data Center web site: http://www.ngdc.noaa.gov/stp/solar/solardataservices.html (National Geophysical Data Center, Solar—Terrestrial Physics Division, Boulder, CO, USA).

The low frequency rhythmicity showed for CBV, TNC and CD34+ progenitor cells, especially the circadecennial pattern, could suggest an association with the circadecennial rhythm of solar fluency, also known as the Schwabe cycle of solar storms. Similarly, the low frequency circannual pattern may link to the circannual rhythm of geomagnetism derived from the Geostationary Operational Environmental Satellites (GOES) data on solar flares, solar proton events and geomagnetic activity (Fig. 4). The pioneering research on the effects of gamma radiation^50^ and geomagnetism^51^ on life forms, as well as the organismic sensitivity to atmospheric electromagnetic forces^52^ has highlighted the space environment influence on living beings. The sun influences all living organisms to some extent, with day/night length changes directly conveying annual time cues to virtually every non-equatorial life-form and circadian time cues to almost all living beings.

Most life-forms are able to read the message conveyed by light, a form of electromagnetic radiation perceived as visible light when the wavelength is between approximately 400 nm and 700 nm, infrared when it is longer than 700 nm, and ultraviolet (UV) when it is shorter than 400 nm. In particular, animals utilize irradiance changes to regulate their internal circadian clocks and some clock neurons are highly sensitive to variations in spectral composition occurring over twilight, with blue-yellow color discrimination affording a more consistent method of tracking twilight progression than simply measuring irradiance^53^.

Furthermore, low frequency non-photic solar rhythms influence human biological time structures, likely mediated via UV irradiation, solar protons and heavy charged particles, as well as geomagnetic storm induced gravitational field changes, fluctuations and resonance signals related to non-photic solar fluency. The possibility that geomagnetic effects related to solar activity may be predominantly involved rather than changes in UV-light is corroborated by reported changes of nocturnal urine 6-sulfatoxymelatonin in untreated female Sprague-Dawley rats during solar cycle 23, beginning in May 1996 and ending in January 2008. These results highlight a possible involvement of non-photic cues and give hypothetical support to a seasonal Zeitgeber function of the horizontal intensity H of the geomagnetic field that shows circannual variations. Additionally, the 11-years’ sunspot cycle also drives geomagnetic disturbances and could facilitate seasonal 6-sulfatoxymelatonin rhythmicity during specific years^54^,^55^. Interestingly, in our study CBV showed a clear trough after the solar maximum (1999–2002), at a time when geomagnetic disturbances were maximal during the last solar cycle. In addition, high TNC content paralleled the solar maximum, declining towards the end of the cycle, showing an opposite pattern respect to CBV time related variation. This latter effect was also apparent in the pattern of variation of CD34+ cells. It is of note that low-frequency temporal patterns have been documented in a variety of physiological measurements, including heart rate, blood pressure, temperature and respiration. Moreover, a 10-year rhythm in birth and death statistics has been reported by population biologists, and infra-annual cycles in heliogeophysical activity may drive the rate of other biological phenomena, including the incidence of pathological conditions, such as cardiovascular events^23^,^56^. At present, our study is the first investigating together potential circadecennial, circannual and circadian variations in cord blood composition. Most studies showed circadian rhythmicity of stem and progenitor cell proliferation and differentiation in the bone marrow in mammals and hinted increase of hematopoietic stem/progenitor cell concentrations in the peripheral blood in the afternoon or in the evening in humans. Accordingly, stem cell yield in healthy adult donors undergoing granulocyte-colony stimulating factor-induced mobilization for allogeneic stem cell donation was dependent on the collection time of day, with higher cell yields obtained when the apheresis was performed in the afternoon rather than in the morning^40^. Regarding the circadecennial rhythmic patterns, our data contribute to supportive analyses hinting an involvement of ten-yearly geomagnetic field variations in the oscillation of biological phenomena as well as the potential participation of cyclic components of heliogeophysical activity such as sunspot seasonality and solar activity rhythmicity in (patho) physiological mechanisms.

In conclusion, the results of our retrospective analysis of data derived from cord blood donations confirm the importance of maternal and neonatal factors associated with stem cell harvesting in UCB units, and have suggested a role for time related changes. That temporal dimensions influence the volume, as well as the content of TNC and CD34+ cells, in UCB, indicates a profound impact of time domains on the dynamics of precursor cell retrieval, with important implications for the amount of stem cell yield from UCB donations. The data of our study relying on near 20000 cord blood samples corroborate circadian rhythmicity of hematopoietic cell trafficking in humans also in pre-natal life and may suggest useful proposals for cord blood banking, pointing out that the temporal dimension of delivery may influence hematopoietic cell harvest in cord blood units and deserves consideration when planning cord blood units collection with the aim to attain the highest hematopoietic potential.

## Additional Information

**How to cite this article**: Mazzoccoli, G. *et al*. Time related variations in stem cell harvesting of umbilical cord blood. *Sci. Rep.* **6**, 21404; doi: 10.1038/srep21404 (2016).

## Supplementary Material

Supplementary Information

## Figures and Tables

**Table 1:** Baseline maternal, newborn and cord blood characteristics.

		Overall	Apulia	Tuscany
Cord blood donations (n)(%)		17936 (100%)	7364 (41.06%)	10572 (58.94%)
Maternal age (year)	Mean ± SD	32.47 ± 4.63	32.45 ± 4.86	32.48 ± 4.45
	Median (min-max)	32.80 (15.00–49.00)	32.73 (15.00–49.00)	33.00 (16.00–49.00)
Route of delivery (n)(%)	Missing values	2024 (11.28%)	730 (9.91%)	1294 (12.24%)
	Vaginal	12600 (70.25%)	4002 (54.35%)	8598 (81.33%)
	Cesarean	3312 (18.47%)	2632 (35.74%)	680 (6.43%)
Gestational age (week)	Mean ± SD	39.39 ± 1.20	39.28 ± 1.20	39.47 ± 1.19
	Median (min-max)	39.50 (35.00–43.00)	39.00 (35.00–43.00)	40.00 (35.00–43.00)
Length of labor (min)	Mean ± SD	253.49 ± 170.71	253.49 ± 170.71	Not available data
	Median (min-max)	225.00 (0.00–1250.00)	225.00 (0.00–1250.00)	
Duration of rupturedmembranes (min)	Mean ± SD	118.13 ± 168.89	118.13 ± 168.89	Not available data
	Median (min-max)	55.00 (0.00–1320.00)	55.00 (0.00–1320.00)	
Placental weight (g)	Mean ± SD	522.64 ± 107.22	522.64 ± 107.22	Not available data
	Median (min-max)	500.00 (170.00–1300.00)	500.00 (170.00–1300.00)	
Newborn weight (g)	Mean ± SD	3361.67 ± 395.87	3350.34 ± 395.91	3370.22 ± 395.65
	Median (min-max)	3340.00 (2200.00–5300.00)	3330.00 (2220.00–5130.00)	3350.00 (2200.00–5300.00)
Apgar score 1 minute	Mean ± SD	8.80 ± 1.24	8.51 ± 0.76	9.01 ± 1.46
	Median (min-max)	9 (1–9)	9 (1–10)	9 (1–10)
Apgar score 5 minutes	Mean ± SD	9.56 ± 0.64	9.39 ± 0.60	9.67 ± 0.64
	Median (min-max)	9 (1–10).	9 (1–10)	9 (1–10)
Newborn sex (n)(%)	Missing values	224 (1.25%)	64 (0.87%)	160 (1.51%)
	Female	8658 (48.27%)	3516 (47.75%)	5142 (48.64%)
	Male	9054 (50.48%)	3784 (51.39%)	5270 (49.85%)
Banking (%)	Missing values	98 (0.55%)	52 (0.71%)	46 (0.44%)
	Not	15518 (86.52%)	5894 (80.04%)	9624 (91.03%)
	Yes	2320 (12.93%)	1418 (19.26%)	902 (8.53%)
Umbilical cord blood volume (CBV) (ml)	Mean ± SD	71.12 ± 28.40	84.25 ± 26.18	62.00 ± 26.24
	Median (min-max)	68.57 (10.00–231.43)	81.00 (19.05–231.43)	59.00 (10.00–214.47)
Total nucleated cells (TNC)	Mean ± SD	983.57 ± 465.89	875.96 ± 436.00	1128.44 ± 465.63
	Median (min-max)	906.67 (10.30–4914.67)	797.95 (36.00–4914.67)	1052.00 (10.30–4359.60)
CD34 + × 10^6^	Mean ± SD	3.51 ± 3.08	3.78 ± 2.85	2.49 ± 3.64
	Median (min-max)	2.90 (0.01–33.27)	3.13 (0.07–30.83)	0.92 (0.01–33.27)
Colony forming units (CFU)	Mean ± SD	88.15 ± 63.96	125.05 ± 65.31	52.40 ± 36.50
	Median (min-max)	71.25 (1.00–550.00)	110.50 (6.05–550.00)	46.50 (1.00–538.17)

**Table 2:** Results from generalized multivariable linear mixed models.

Covariates	CBV	TNC	CD34 × 10^6^
Year of birth	*p* < 0.001 (R^2^ = 0.022)	*p* < 0.001 (R^2^ = 0.028)	*p* < 0.001 (R^2^ = 0.043)
Month of birth	*p* = 0.390 (R^2^ = 0.000)	*p* = 0.399 (R^2^ = 0.002)	*p* = 0.021 (R^2^ = 0.005)
Day of birth	*p* = 0.403 (R^2^ = 0.002)	*p* = 0.501 (R^2^ = 0.009)	*p* = 0.653 (R^2^ = 0.010)
Hour of birth	*p* = 0.153 (R^2^ = 0.002)	*p* = 0.557 (R^2^ = 0.009)	*p* < 0.001 (R^2^ = 0.035)
Maternal age	*p* = 0.542 (R^2^ = 0.001)	*p* = 0.629 (R^2^ = 0.002)	*p* = 0.951 (R^2^ = 0.001)
Gestational age	*p* < 0.001 (R^2^ = 0.005)	*p* < 0.001 (R^2^ = 0.005)	*p* = 0.001 (R^2^ = 0.012)
Route of delivery	*p* < 0.001 (R^2^ = 0.013)	*p* < 0.001 (R^2^ = 0.014)	*p* = 0.182 (R^2^ = 0.002)
Newborn sex	*p* < 0.001 (R^2^ = 0.002)	*p* < 0.001 (R^2^ = 0.006)	*p* = 0.936 (R^2^ = 0.003)
Newborn weight	*p* < 0.001 (R^2^ = 0.085)	*p* < 0.001 (R^2^ = 0.557)	*p* < 0.001 (R^2^ = 0.019)
Total R^2^	R^2^ = 0.135	R^2^ = 0.631	R^2^ = 0.129

P-values from type III test for fixed effects and, in round brackets, partial R^2^ coefficients for goodness of model fit. Total R2 coefficients measure the overall goodness of fit of the statistical model to observed data. Partial R2 coefficients measure the marginal contribution of each covariate on the total variability of the outcome, when all other covariates are already included into the model. Total and partial R2 coefficients range from 0 (poor model fitting) and 1 (excellent model fitting).

**Table 3:** Parameters estimated by cosinor models fitted on adjusted means of cord blood volume (CBV), total nucleated cells (TNC) and CD34 + progenitor cells.

*Best-fit curves (Period)*	*Parameter*	CBV (log-ml)		TNC (log-counts)		CD34+ × 10^6^ (log-counts)	
		*Estimate* ± *SE*	*p-value*	*Estimate* ± *SE*	*p-value*	*Estimate* ± *SE*	*p-value*
10 years for CBV, TNC and CD34 + cells	MESOR	4.154 ± 0.018	<0.001	6.967 ± 0.025	<0.001	1.368 ± 0.073	<0.001
	Semi-Amplitude	0.133 ± 0.007	<0.001	0.108 ± 0.009	<0.001	0.388 ± 0.038	<0.001
	Acrophase	0.876 ± 0.064	<0.001	3.235 ± 0.072	<0.001	3.549 ± 0.073	<0.001
6 months for CBV, TNC 12 months for CD34 + cells	MESOR	4.154 ± 0.014	<0.001	6.967 ± 0.019	<0.001	1.368 ± 0.039	<0.001
	Semi-Amplitude	0.004 ± 0.004	0.250	0.008 ± 0.006	0.169	0.041 ± 0.013	0.001
	Acrophase	3.801 ± 1.691	0.025	3.369 ± 1.234	0.006	7.149 ± 0.899	<0.001
15 days for CBV, TNC 1 month for CD34 + cells	MESOR	4.154 ± 0.021	<0.001	6.967 ± 0.028	<0.001	1.368 ± 0.058	<0.001
	Semi-Amplitude	0.005 ± 0.004	0.232	0.015 ± 0.006	0.015	0.036 ± 0.013	0.005
	Acrophase	0.885 ± 2.378	0.710	1.496 ± 1.651	0.365	4.275 ± 1.220	<0.001
24 hours for CBV, CD34 + cells 8 hours for TNC	MESOR	4.154 ± 0.019	<0.001	6.967 ± 0.026	<0.001	1.368 ± 0.057	<0.001
	Semi-Amplitude	0.015 ± 0.005	0.004	0.023 ± 0.007	0.001	0.080 ± 0.016	<0.001
	Acrophase	4.196 ± 1.035	<0.001	7.295 ± 0.738	<0.001	7.636 ± 0.981	<0.001

Cosinor = least-squares fit of a single component cosine curve to all data. MESOR = rhythm-adjusted overall mean for log-transformed parameters; Amplitude = peak-trough range of cosine model; Acrophase (radians) = peak of cosine in radians; *p*-value = *p* value for statistical significance of parameter estimates: rhythm detection was considered statistically significant when p < 0.05.
