# Supplementary material for: Time related variations in stem cell harvesting of umbilical cord blood
Source: Sci Rep. 2016 Feb 24;6:21404. doi: 10.1038/srep21404 (PMC4764902; doi:10.1038/srep21404)

**Time related variations in stem cell harvesting of umbilical cord blood**

Gianluigi Mazzoccoli^1†*^, Giuseppe Miscio^2†*^, Andrea Fontana^3†^, Massimiliano Copetti^3^, Massimo Francavilla^4^, Alberto Bosi^5^, Federico Perfetto^6^, Alice Valoriani^7^, Angelo De Cata^1†^, Michele Santodirocco^2^, Angela Totaro^2^ , Rosa Rubino^1^, Lazzaro di Mauro^2†^, Roberto Tarquini ^7†^

^1^ Department of Medical Sciences, Division of Internal Medicine and Chronobiology Unit, IRCCS “Casa Sollievo della Sofferenza”, S.Giovanni Rotondo (FG), Italy

^2^ Apulia Cord Blood Bank, IRCCS “Casa Sollievo della Sofferenza”, S.Giovanni Rotondo (FG), Italy

^3^ Unit of Biostatistics, IRCCS “Casa Sollievo della Sofferenza”, S.Giovanni Rotondo (FG), Italy

^4^ Computing Unit, IRCCS Scientific Institute and Regional General Hospital “Casa Sollievo della Sofferenza”, S.Giovanni Rotondo (FG), Italy

^5^ Department of Clinical and Experimental Medicine, Unit of Haematology, School of Medicine, University of Florence, Florence, Italy

^6^ Department of Clinical and Experimental Medicine, School of Medicine, University of Florence, Florence, Italy

^7^ Interinstitutional Department for Continuity of Care of Empoli, School of Medicine, University of Florence, Florence, Italy

^†^ These authors contributed equally to this article

Supplemental Data: Figures S1-S3

Supplemental Figure 1. *x-y* plots showing from top to bottom the variation patterns in different time domains of raw means (ovals) of volume in the cord blood donations in Tuscany and Apulia Cord Blood Banks from 1999 to 2011. Error bars represent standard deviation (SD) around the raw means (i.e. mean±SD)

Supplemental Figure 2. *x-y* plots showing from top to bottom the variation patterns in different time domains of raw means (ovals) of total nucleated cells (TNCs) in the cord blood donations in Tuscany and Apulia Cord Blood Banks from 1999 to 2011. Error bars represent standard deviation (SD) around the raw means (i.e. mean±SD)

Supplemental Figure 3. *x-y* plots showing from top to bottom the variation patterns in different time domains of raw means (ovals) of CD34+ progenitor cells in the cord blood donations in Tuscany and Apulia Cord Blood Banks from 1999 to 2011. Error bars represent standard deviation (SD) around the raw means (i.e. mean±SD)


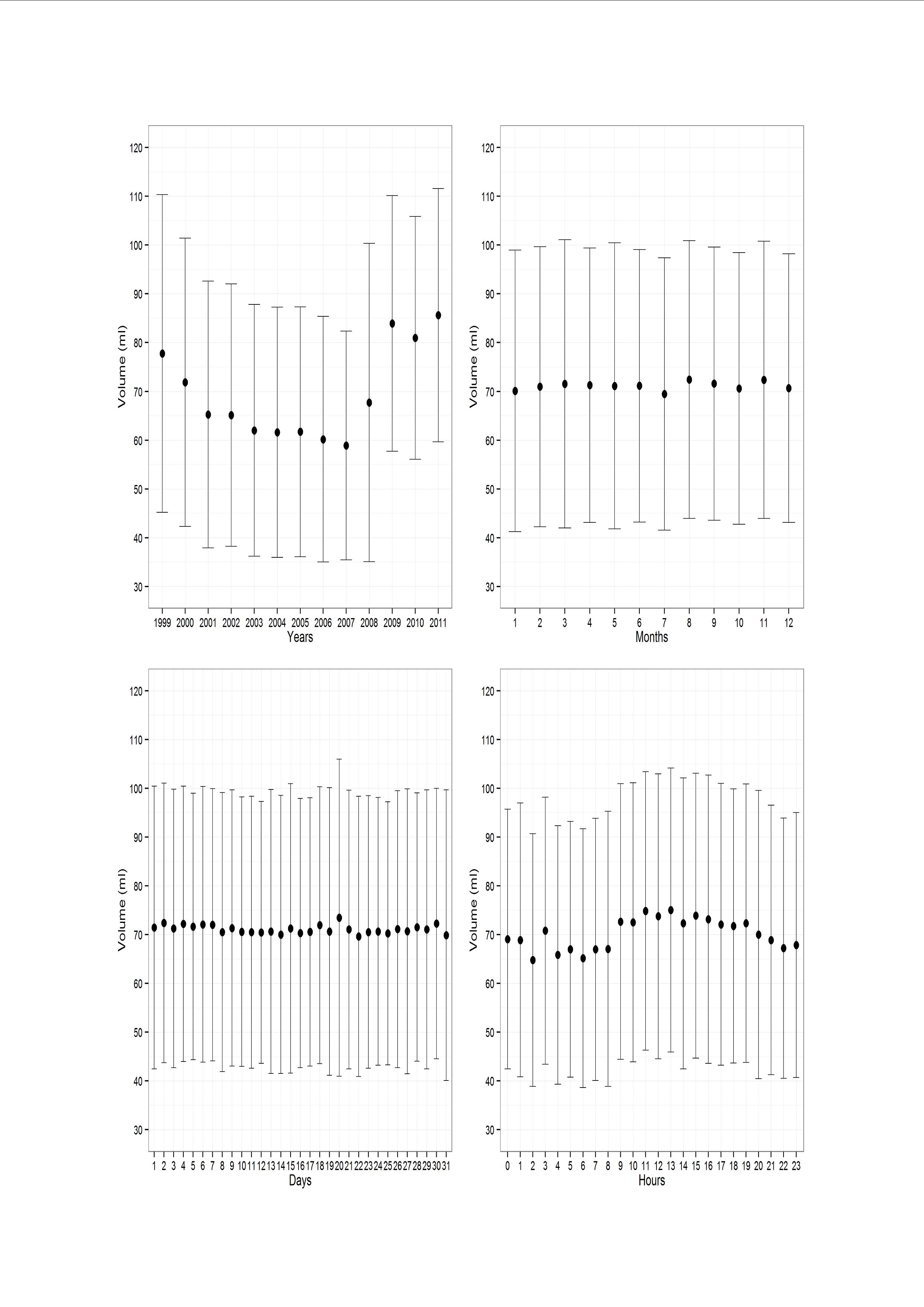


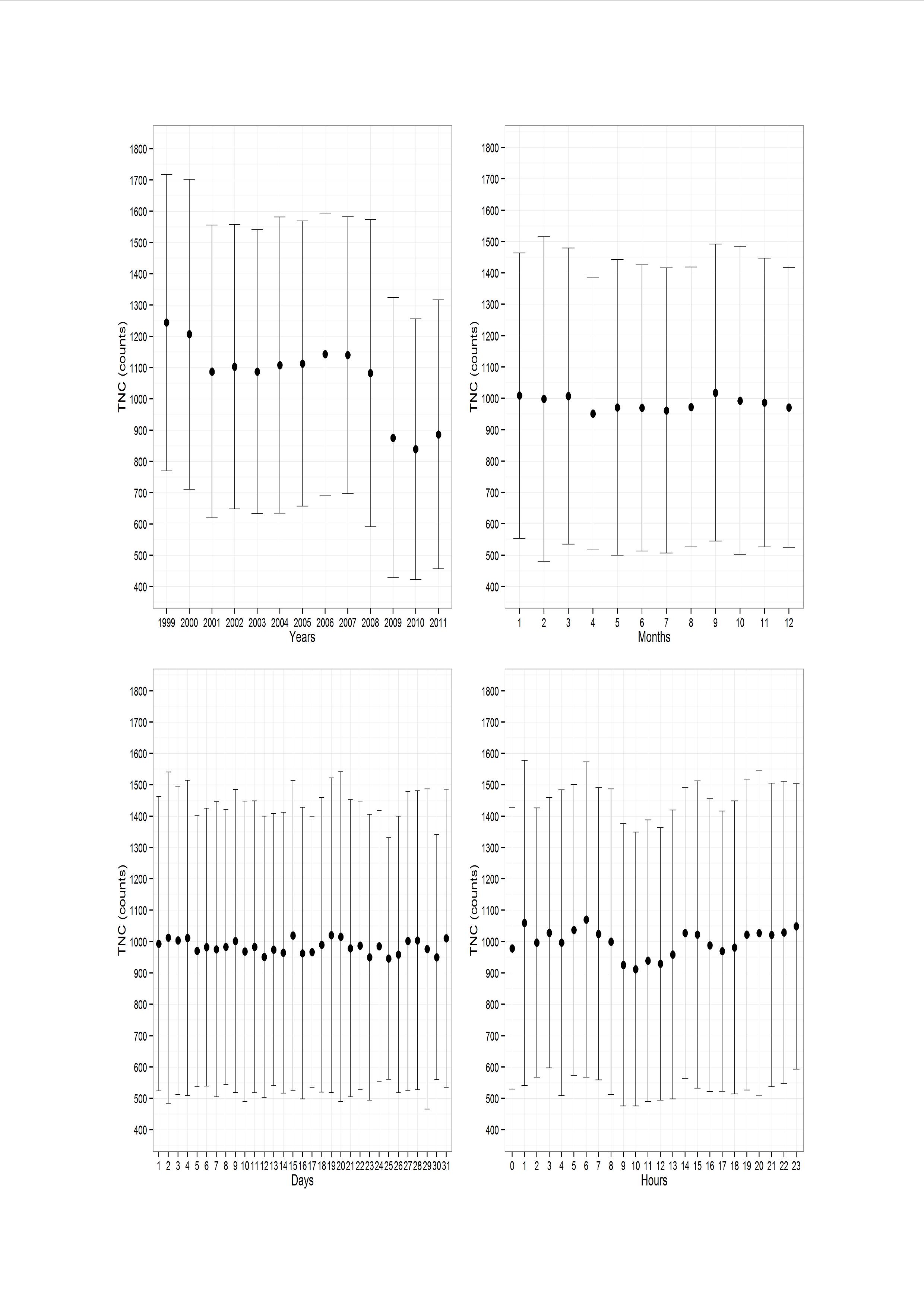


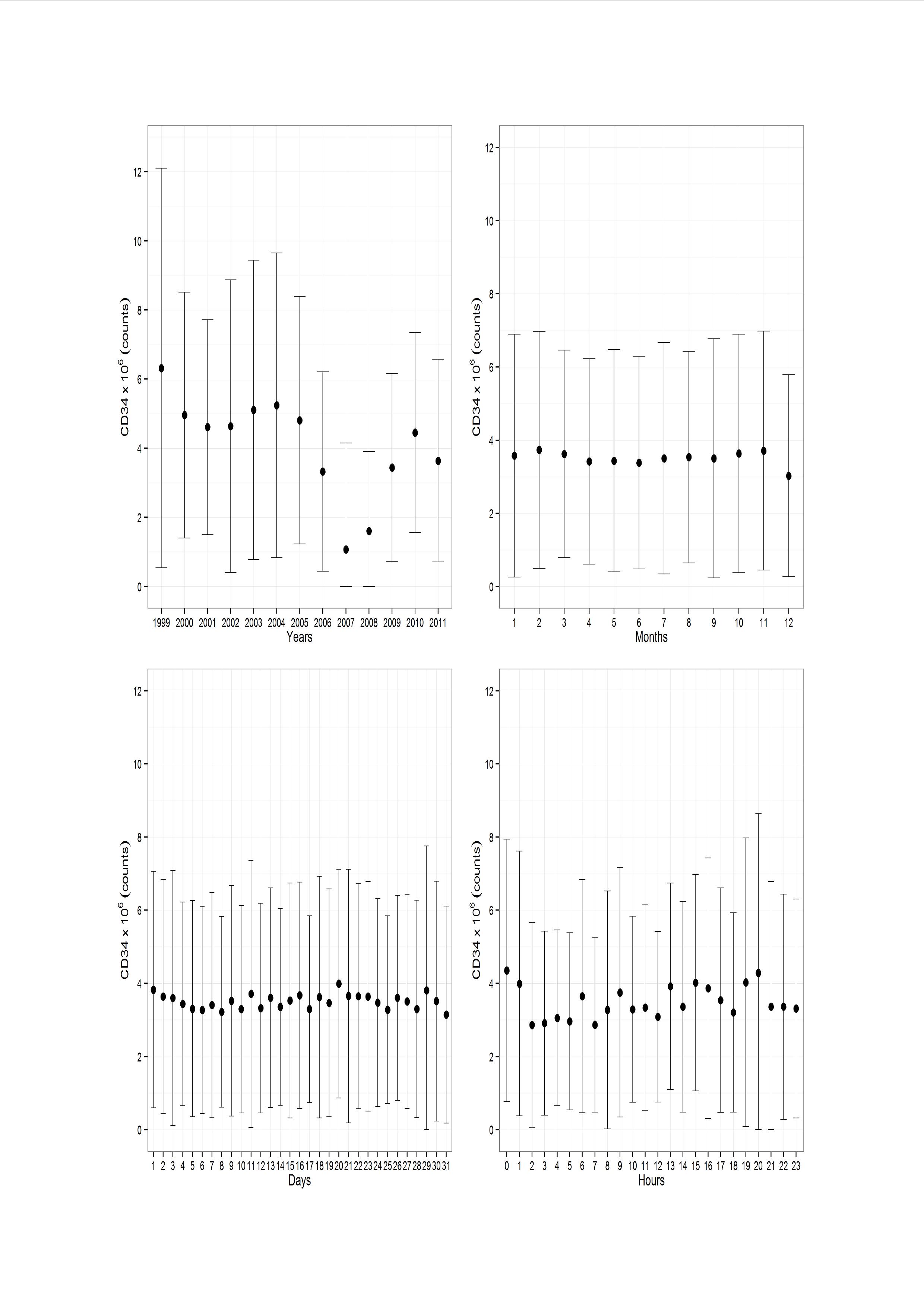

Supplement: Supplementary Information [file srep21404-s1.doc]
